# Barriers to participating in an online family- and group-based prevention programme for parents with depression: an online survey

**DOI:** 10.1186/s40359-024-02266-8

**Published:** 2025-03-19

**Authors:** Angela Joder, Svenja Geissler, Petra Dengl, Gerd Schulte-Körne, Belinda Platt

**Affiliations:** Department of Child and Adolescent Psychiatry, Psychosomatics and Psychotherapy, LMU University Hospital, Munich, Germany

**Keywords:** Implementation, Video-conferencing, Digital, Mixed-method, Intervention, Preventive

## Abstract

**Background:**

Children of parents with depression have an increased risk of mental illness themselves and there is an urgent need to implement effective prevention programmes for this population. "Growing Up Healthy and Happy" (“GuG-Auf-Online") is an online family- and group-based cognitive-behavioural preventive programme with a strong evidence base. The aim of the current study was to understand what factors might hamper parents with depression from participating in the programme.

**Methods:**

An online cross-sectional survey was conducted in Germany with 274 parents who fulfilled the inclusion criteria for the programme (parental history of depression and a child aged eight to 17 years with no mental illness). The survey included several a priori-defined barriers (e.g. online format, feelings of shame) which parents rated in terms of (a) whether the barrier was relevant to them and if so, (b) how much it held them back from participating. Open-ended questions identified additional barriers. In addition to qualitative content analysis according to Mayring (2008), Pearson correlations were calculated to determine whether the current severity of parents’ symptoms were associated with their responses.

**Results:**

The following aspects emerged as relevant barriers: (a) shame regarding one's depression, (b) overburden and (c) avoidance (not wanting to be reminded of depression). There was no evidence that the online setting was a significant barrier. Most of the correlations between the current severity of parent’s symptoms and their responses were statistically significant (*p* < .0037).

**Conclusions:**

The main barriers to participation in prevention related to individual characteristics/ emotional experiences rather than structural issues. Addressing these barriers in the advertisement of future programmes could improve uptake.

**Supplementary Information:**

The online version contains supplementary material available at 10.1186/s40359-024-02266-8.

## Introduction

Children of parents with a history of depression represent a particularly high-risk group for developing mental illness: approximately 58% of these children will experience an episode of mental illness during their own lifetime [30]. Compared to peers with parents who have not experienced depression, they are up to three times more likely to develop a depressive disorder during childhood and adolescence [24, 32] and the increased risk continues into adulthood [12, 31]. Children with depression are likely to have difficulties with friendships, social interactions, and school performance, and may be impaired in their social, emotional, and cognitive development [3, 27]. Adolescent depression is also associated with a high risk of suicide [12], comorbid mental health disorders (e.g., anxiety disorders, aggressive behavior, substance use disorders [27], and risk of mental illness in adulthood [12, 31]. Implementing effective prevention programmes for mental disorders is a public health priority [33].

Studies show that psychological programmes for children of parents with depression who themselves have no history of mental disorder significantly reduce the child’s risk of depression [17]. Prevention programmes for this target group address the challenges these families face by (a) providing child-oriented psychoeducation about depression, (b) teaching children coping strategies for dealing with stressors known to trigger a depressive episode, and/or (c) supporting adaptive parenting techniques [17]. The Family- and Group-based Cognitive-Behavioral (FGCB) intervention addresses all three elements and has demonstrated positive effects on depression risk compared to a control condition [6]. The FGCB was adapted to German language and culture ("Grow Up Healthy and Happy" or "GuG-Auf" in German language) and the positive effects replicated in a recent RCT by the current authors [16, 18]. GuG-Auf addresses the prevention of psychopathology in children and includes sessions that, depending on the content discussed, take place a) with children, b) with parents and c) with both children and parents. Whilst the evidence-base for GuG-Auf is relatively high, the suitability of the intervention for implementation in the healthcare system is questionable. Firstly, families who participated in the initial trial had relatively high socioeconomic status, suggesting it was less attractive for families with fewer financial resources [18]. Secondly, participating families described the intervention as too time-consuming [5], suggesting that families with less time might have been put off from taking part. To facilitate access to the intervention and reduce face-to-face contact during to the covid-19 pandemic, an online version of the intervention (GuG-Auf-Online) was developed and is currently being evaluated [26]. In GuG-Auf-Online the number of sessions was reduced from twelve to eight and the delivery via video conferencing enabled families outside of the local area to access it. The effectiveness of digitally-delivered evidence-based interventions has been demonstrated for both adults [2] and adolescents [8]. However, despite numerous attempts to make the GuG-Auf-Online intervention more appealing to a wider-range of parents with depression, recruitment to the trial proved difficult: within the first year (July 2020 to October 2021), only five eligible families were randomized, although by November 2022 this number had risen to 37. Whilst it is possible that the burden families faced by the covid-19 pandemic contributed to poor recruitment, similar effects have been reported in other trials of prevention programmes. One review of prevention studies found that recruitment often spans several years and, on average, only about 40% of families who were contacted and met eligibility requirements participate in the preventive intervention [10]. In one case, an RCT study was discontinued due to low numbers of participating families [9]. If evidence-based interventions for parents with depression are to be implemented effectively, more needs to be understood about the barriers these parents face.

A handful of studies have investigated the barriers to participate in prevention facing parents with a history of depression [9, 25, 28]. Some parents report feeling too burdened by their symptoms and comorbid mental disorders or fear that they would be further burdened by the time and effort needed for study and participation in a prevention programme [9]. Other parents who are currently in remission express a desire not to be reminded of their depression [9, 28] or do not see a need for prevention given their current situation [9]. Some parents fear that participating in the intervention may be too confrontational [9] or may identify something they have done wrong [25]. Other parents have intense feelings of shame and guilt [9, 25] and/or fear of stigmatization [9, 25]. Some parents report that they believe their children are too young to participate in an intervention [9] and that they want to protect their children from possible negative effects of participation [9, 25]. In addition, some parents indicate their children are not motivated to participate in an intervention [9, 25]. Furthermore, some parents do not want to talk to their children about mental disorders but do not explain why [28]. Although these studies point towards a number of barriers facing parents with depression, they are also limited methodologically and in terms of content. For example, the sample sizes are relatively small (*N* = 24; [9]) or not explicitly stated [25, 28]. Furthermore, none have evaluated barriers to interventions for multiple families or to an online intervention. Moreover, no study has examined whether there is an association between current depression severity and participation in a prevention programme. In summary, the literature so far is limited and a large survey of parents with depression which comprehensively addresses a wide range of barriers to participation in online prevention is a necessary next step.

### The current study

The overarching goal of the current study was to identify the possible barriers parents with a history of depression face to participating in the GuG-Auf-Online prevention programme. The study was also intended to inform the implementation of family-, group- and/or online-based preventive interventions more generally. Due to the limitations of previous studies described above, a research-generating rather than hypothesis-testing approach was taken. Barriers which arose from the previous studies were included in the survey and complemented by barriers relevant to the GuG-Auf-Online prevention programme specifically (e.g. the online setting, the impact of the covid-19 pandemic) as well as barriers which we predicted would play a role given our clinical experience with parents with depression. Specifically, the following research questions regarding the participation in GuG-Auf-Online were addressed:

1. Which barriers with regard to participation in GuG-Auf-Online do parents frequently report being relevant to them in some way? Which barriers are less frequently reported?
2. To what extent do the various barriers hinder parents from taking part in the prevention programme?
3. Is the impact of a given barrier associated with current parental depression severity?

In addition, participants were also able to provide open-ended responses about additional barriers to participation that were not previously listed.

## Method

### Study Design

This cross-sectional online survey was conducted between November 2021 and June 2022 via LimeSurvey. Since many aspects that would prevent parents from participating in GuG-Auf-Online could also prevent them from participating in the survey about their barriers, a number of methodological decisions were made to minimise barriers to survey participation. Firstly, the online format was chosen to promote anonymity since parents may fear prejudice, disclosing personal information about their family and/or reluctance to be confronted with their weaknesses. To maximise anonymity no personally-identifiable data (e.g. age in years, city of residence) were collected. Where possible, wording which was non-stigmatising and understandable to parents who were less educated and/or those with different cultural backgrounds was used. Due to the burden faced by parents with depression the length of the survey was kept to a minimum by including predominantly fixed-choice answers. Due to the frequent underrepresentation of parents with lower socioeconomic status in prevention programmes adequate renumeration for parents’ time was provided (€15 for roughly 15 min).

The inclusion criteria for the online survey were based on the inclusion criteria for GuG-Auf-Online, but could not always be operationalized tightly due to the brief online format. To include parents who had likely experienced depression but had not necessarily been diagnosed by a mental health professional, parents were included who described themselves as having “experience of symptoms of depression such as prolonged sadness and exhaustion”. In order to maximize recruitment participants were not required to state whether an episode had occurred during the child’s lifetime or prior to the child’s birth. Parents were included if they had a child between 8 and 17 years of age who they themselves identified as not having experienced mental illness. In contrast to Festen et al. [9], no non-affected partners were included, as the focus of the research question was on the perspective of the parent with depression.

### Sample

*N* = 339 individuals participated in the survey. Several individuals (*n* = 65) were excluded from data analysis: *n* = 34 participants did not have a child in the relevant age range, *n* = 28 participants did not provide information on the age of their children, and *n* = 3 participants wrote in open-ended responses that they did not belong to the target group. Thus, the data from *n* = 274 parents were analysed.

### Procedure of the online survey, instruments and items

The survey consisted of several components, which are presented below.

#### Information text about symptoms of depression and the survey

We gave brief information about our definition of depression symptoms (for parents without a confirmed diagnosis) and informed them about the ethical and data protection aspects of the study, the study duration (15 min) and reimbursement (€15 voucher). Participants then provided informed consent to participate.

#### Assessment of barriers to participation in GuG-Auf-Online

A complete overview of all items assessing the barriers can be found in Table 1.

**Table 1:** Items used to capture potential barriers and their overarching themes

Overarching theme	Item wording
*(a) Online, family and group settings*	
Do not want to disclose	^a^I do not want to talk to or open up in front of my child's father/mother
	^a^I do not want to open up in front of my child or children
	^a^I do not want to open up in front of other families
Additional internet usage	^a^I don't want my children to spend more time online than they already do
*(b) Emotional concerns of parents*	
Burden	I feel too burdened by my depression
	^a^I feel too burdened by the covid-19 pandemic
Guilt	I have feelings of guilt
	I want to avoid developing feelings of guilt
Shame	I feel ashamed of my depression
Not feeling depressed anymore	I dont’t feel depressed anymore
Not being reminded of depression	I don't want to be constantly reminded of my depression
*(c) Linking of emotional concerns and the setting*	
Fear of prejudice	^a^I am afraid that the other participants or the therapists involved will be prejudiced against me
Confrontation with own weaknesses	^a^I am afraid that I will be confronted with my own weaknesses during the study
Exposure of violence/neglect	^a^I am afraid that during my participation in the study it will become known that my children experience violence or are neglected by me
Negative effects	I am concerned about negative effects on my child or children
*(d) Others*	
Do not accept support	I do not want to receive psychotherapeutic support (anymore)
	I would never accept psychotherapeutic support
No need	My child/children do not need support
	I see no connection between my mental health and the mental health of my child/children
Doubts to meet inclusion criteria	^a^My child is already receiving psychotherapy treatment
	^a^I have never been diagnosed with depression
Culture	In my culture, no one would participate in a prevention program like this
	^bc^ Are there any reasons that might prevent members of your cultural group from participating?
Motivation child	My child or children refuse to participate
Child too young	I think my child or children are too young to participate
Lack of information	^a^I have not received enough information about the program
Time spent	The time commitment is too much for me
Doubts about efficacy	^a^I doubt that prevention can help
Additional barriers	^b^ Are there any other reasons or possible barriers that might prevent you from participating in the study?

*Notes.* Introductory text: "For each statement, please indicate how much it discourages you from participating. If the statement does not apply to you, please check "not relevant to me.""

^a^ These items are based on expert considerations. ^b^ These items were open-response format items that did not require a response. ^c^ This item was only displayed if the previous item was not answered in the negative

The selection of barriers was made based on findings from previous studies as well as experiences the study team had gained during recruitment for the trial (see notes in Table 1). Since the purpose of assessing barriers was not to record the expression of concrete constructs (e.g., guilt, shame) and since no standardized questionnaire could be found that captured such barriers in regard to participation in a prevention programme, the items were self-constructed. The items considered a variety of aspects, such as barriers due to the online, family, and group setting, barriers due to parents' emotional concerns, barriers linking emotional concerns and the setting, and other barriers. Participants were able to indicate whether a barrier did not apply to them at all (not relevant to me [0]) or to what extent that barrier kept them away from participating in GuG-Auf-Online, if it applied (does not keep me away [1], keeps me away a little [2], keeps me away moderately [3], keeps me away quite a bit [4], and keeps me away a lot [5]). For example, if survey participants did not identify (exclusively) with the German culture, their culture could be a potential barrier to participation (e.g. because their culture hardly ever talks openly about psychological stress).

Nevertheless, their culture might not necessarily prevent them from taking part in the prevention programme (“does not keep me away”), e.g. because the person still talks openly about psychological stress. In two additional questions with an open answer format, further barriers could be named.

#### Assessment of symptoms

A German version of the Depression Anxiety Stress Scales (DASS; [23]) was used to assess depression symptom severity in the past 7 days. The inventory consists of 21 items assigned to 3 subscales: *Depression Scale*, *Anxiety Scale*, *Stress Scale*. The items are scaled on four levels from did not apply to me at all (0) to applied to me very much or most of the time (3). The depression scale correlates moderately (*r* = 0.56) with the Beck Depression Inventory [23]. In addition, several self-constructed items were used. These items concerned current symptom severity ("Do you currently feel depressed?" and "How long have you felt depressed?" and "How long have you not felt depressed?" respectively) and the number of previous depressive episodes ("How many episodes of depression have you experienced in total in your life?").

#### References to contact points in emergency situations

References to psychiatric clinics and crisis centres in Germany were provided, in case parents experienced acute psychological distress at the time of participation.

#### Assessment of sociodemographic characteristics

Self-constructed items were used to record age, gender, educational attainment, number of inhabitants in the place of residence, the number and age of children, and current relationship status. In addition, the means by which participants were made aware of the online survey was documented. To allow participants the greatest possible anonymity, the age of the parents was divided into age ranges.

### Recruitment

The flyer for the online survey contained information about GuG-Auf-Online as well as the invitation to participate in the online survey if parents did not intend to participate in GuG-Auf-Online. The flyer was designed in collaboration with an affected mother who encouraged the use of more simple (non-technical, non-judgemental) language. Recruitment for the online survey lasted 7.5 months from early November 2021 to mid-June 2022. Following the recommendations of Havinga et al. [10], several recruitment strategies were followed. These included a nationwide advertisement on Facebook (1 week), a Germany-wide self-help group on Facebook for parents with depression, practices of psychotherapists and psychiatrists, e-mail distribution lists and newsletters for people interested in research, relatives of people with mental illness, and networks for parents. In addition, parents with depression who had approached the research group in the past and had chosen not to participate in the prevention programme were invited to take part in the online survey. Parents with depression were encouraged to share the flyer with other parents via social networks and in their private circles. From November 2021 the adverts for GuG-Auf-Online were also modified to include a link to the online survey. For example, a random selection of parents living in Munich with children aged 8–17 years whose postal addresses were supplied by the local council were sent information about GuG-Auf-Online and the online survey. The majority of participants who took part in the online survey (75%) were recruited through social media and network email distribution lists. Other successful recruitment methods were: adverts in practices and clinics (12%), personal approach by (a) psychotherapist (3%), (b) study team around GuG-Auf-Online (3%), and friends/family/acquaintances (7%).

### Statistical analysis

Descriptive and statistical analyses were carried out using SPSS 26 (IBM Corp., Released [13]). To answer question 1 (Which barriers do parents frequently report being relevant to them in some way? Which barriers are less frequently reported?), frequencies for each barrier were counted and tabulated. To answer question 2 (To what extent do the various barriers hinder parents from taking part in the prevention programme?) frequencies, means, and standard deviations were calculated and tabulated. The open-ended questions about other barriers were analysed using qualitative content analysis according to Mayring [20]. To be able to categorize the written text material, a category system with anchor examples was developed inductively. For example, one category reads “additional appointments undesirable for the child” and one anchor example reads "my child already has enough appointments." The complete category system can be found in the online supplementary material. The smallest coding unit was individual words. Only statements that were substantively related to the research question were categorised. All other statements (e.g., the desire for advice on how to deal with the child) were not categorised. Each statement was assigned to just one category. Some responses were difficult or impossible to understand in writing without further information and questions (e.g., "religion" and "burden too great") and were categorized as not understandable. Categorization was performed by AJ and HC. The inter-coder reliability (κ) was 0.83 (near perfect [15],).

To answer question 3 (Is the impact of a given barrier associated with current parental depression severity?), Pearson correlations were computed between the depression subscale sum score and the impact score of each potential barrier (Likert-scale 1 to 5). When calculating whether correlations differed significantly from zero a Bonferroni correction was applied since 27 tests were conducted in total.

After completing data analysis we realised that it would be possible to descriptively compare the demographic and clinical characteristics of the online survey sample with those who had participated in the intervention. Since the sample size of the GuG-Auf-Online participants is modest, no apriori predictions were made and the two samples were recruited during overlapping but not identical time periods, we decided to descriptively report these comparisons rather than performing statistical analyses.

## Results

### Sample characteristics

**Fig. 1:**
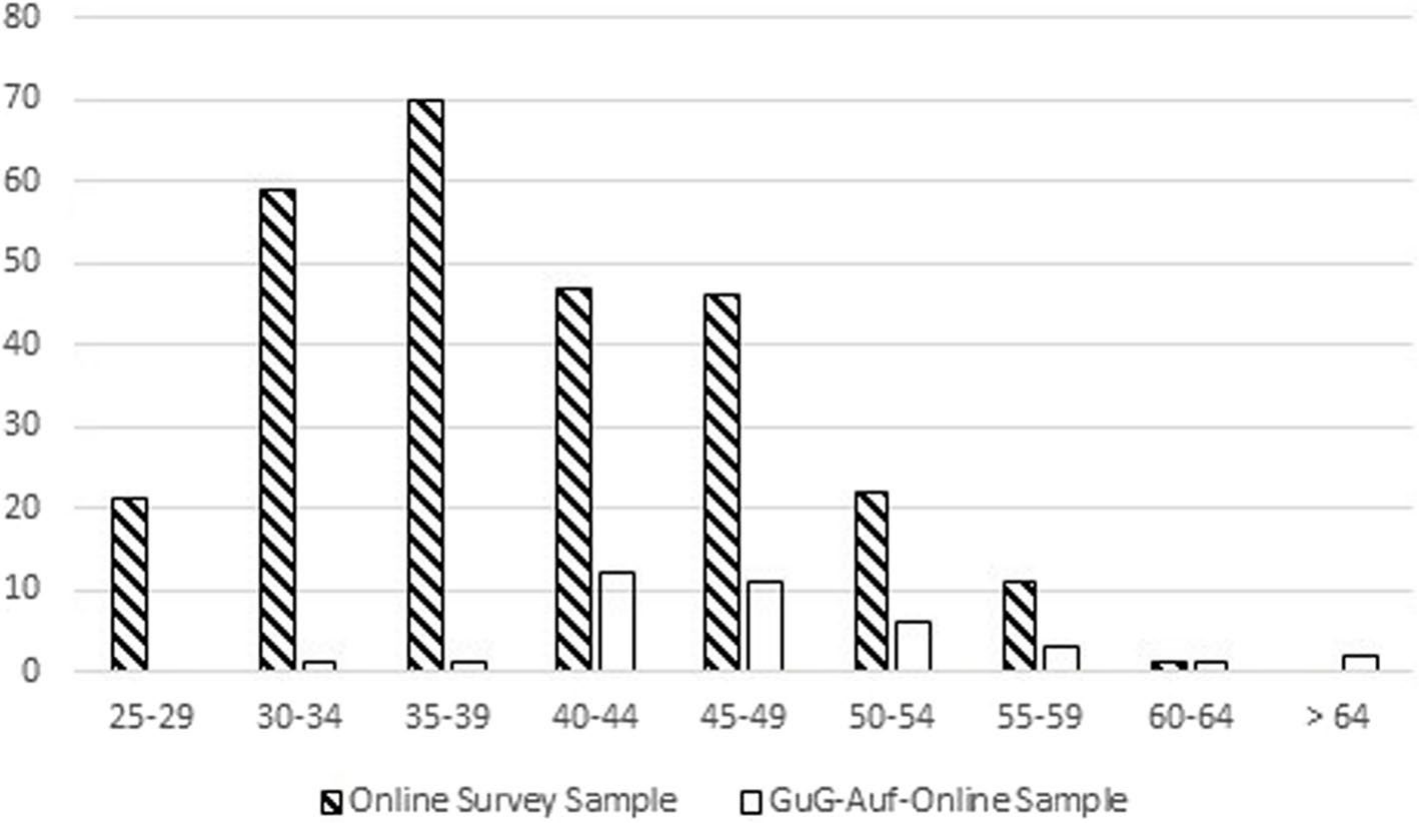
Representation of the age categories of the participants in the online survey and GuG-Auf-Online

**Fig. 2:**
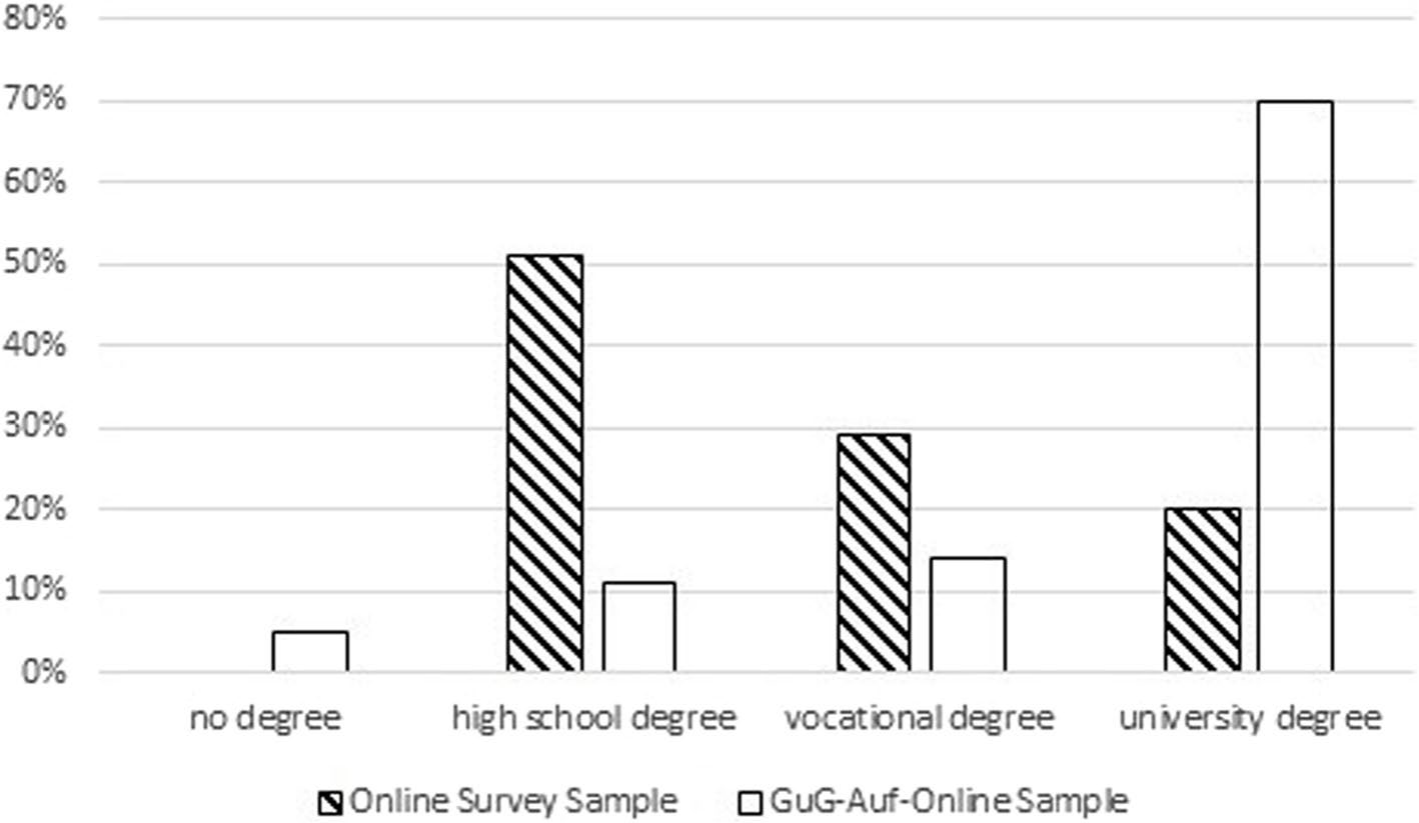
Representation of the highest educational degree of the participants in the online survey and GuG-Auf-Online

Almost a third (32%) of the survey participants were male, 68% female, 0% diverse. The age and education-level of the participants are depicted in Figs. 1 and 2 respectively. Survey participants had an average of *M* = 1.33 children and 77% lived in a partnership. In total, 63% of the survey participants lived in a large city (at least 100,000 inhabitants), 20% in a medium-sized city (20,000 to 100,000 inhabitants), and the remaining participants came from small towns or rural communities.

Online survey participants reported *Mdn* = 3 episodes of depression in their lifetime. In total, 82% of respondents felt depressed at the time of the participation in the online survey (for *Mdn* = past 2 years) whereas 18% did not (for *Mdn* = past 3 years). Scoring of the depression subscale from the DASS inventory suggested that—as mentioned above – only 64% of the sample were above the suggested clinical cut-off (> 10 points).

### Barriers to participation in GuG-Auf-Online

Table 2 shows (a) how many survey participants thought each barrier was relevant or irrelevant to them, (b) to what extent participants were deterred from participating in the prevention programme by each barrier (levels 1 to 5 of the Likert scale) and (c) how many parents were kept away from participating by the barrier (levels 4 and 5 of the Likert scale: keeps me away quite a bit or a lot).

**Table 2:** Descriptive statistics on barriers to participation

	*"Barrier is relevant for me"%*^*a*^ r	*"Barrier kept me away"%*^*b*^ k	*M*	*SD*
I do not want to open up in front of other families	89	8.03	3.33	1.31
I feel ashamed of my depression	91	7.30	3.43	1.21
I don't want to be constantly reminded of my depression	88	7.30	3.45	1.22
I am afraid that I will be confronted with my own weaknesses during the study	89	7.30	3.29	1.25
I feel too burdened by the covid-19 pandemic	93	6.93	3.15	1.32
I feel too burdened by my depression	93	6.93	3.20	1.25
The time commitment is too much for me	96	6.57	2.95	1.38
I do not want to open up in front of my child or children	88	5.11	2.86	1.41
I am afraid that the other participants or the therapists involved will be prejudiced against me	88	4.74	2.85	1.30
I don't want my children to spend more time online than they already do	95	4.38	2.88	1.23
My child or children refuse to participate	77	4.38	2.74	1.39
I am concerned about negative effects on my child or children	84	4.38	2.94	1.23
I have feelings of guilt	89	4.01	2.87	1.25
I want to avoid developing feelings of guilt	79	2.92	2.73	1.22
I doubt that prevention can help	71	2.92	2.68	1.19
I see no connection between my mental health and the mental health of my child/children	73	2.55	2.61	1.19
I do not want to talk to or open up in front of my child's father/mother	80	2.55	2.61	1.24
My child/children do not need support	70	2.55	2.37	1.27
I am afraid that during my participation in the study it will become known that my children experience violence or are neglected by me	56	1.82	2.55	1.26
In my culture, nobody would participate in a prevention program like this	28	1.82	2.95	1.38
I think my child or children are too young to participate	72	1.46	2.41	1.14
I do not want to receive psychotherapeutic support (anymore)	48	1.09	2.29	1.22
I have not received enough information about the program	56	1.09	2.15	1.13
I don't feel depressed anymore	21	0.36	2.11	1.11
I would never accept psychotherapeutic support	52	0.36	1.99	0.99
My child is already receiving psychotherapy treatment	42	0.36	2.26	1.08
I have never been diagnosed with depression	16	0.36	2.13	1.08

*Notes.* Response format: 5-point Likert scale from doesn't keep me (1) to keeps me away a lot (5). The results are

sorted in descending order by n ("Barrier kept me away")

^a^ “Barrier is relevant for me” means that the barrier applies to the participants

^b^ “Barrier kept me away” means that participants answered “keeps me away quite a bit” (4/5) or “keeps me away a lot” (5/5)

*1) Which barriers do parents frequently report being relevant to them in some way? Which barriers are less frequently reported?* It is noticeable that there were numerous and varied barriers that were frequently reported, e.g., (a) time commitment, (b) parents did not want their children to spend more time online than they already do, (c) parents felt too burdened by their depression, (d) the covid-19 pandemic and (e) they felt ashamed of their depression. By far the least frequently mentioned barriers were (a) nobody in my culture would participate in a prevention program like GuG-Auf-Online, (b) parents no longer feel depressed and (c) parents had never been diagnosed with depression.

*2) To what extent do the various barriers hinder parents from taking part in the prevention programme?* Overall, none of the barriers had a particularly high mean value (Min = 1.99, Max = 3.45) or were mentioned rarely (Min = 45, Max = 263). The barriers that keep most parents from participating were: (a) not wanting to open up in front of other families, (b) feelings of shame due to their own depression, (c) not wanting to be reminded of their own depression, (d) fear of being confronted with their own weaknesses, (e) burden of the covid-19 pandemic, (f) burden of depression, and (g) time commitment. In addition to being mentioned frequently, these barriers also had the highest mean scores (2.95—3.33, that means parents were hindered by these barriers moderately). Barriers that less frequently prevented parents from participating were: (a) parents have never been diagnosed with depression, (b) child already receiving psychotherapeutic treatment, (c) parent would never seek psychotherapeutic support, and (d) parent no longer feels depressed.

In two open questions (listed Supplementary Tables 2 and 3), additional barriers of the participants were assessed. This concerned culture-related barriers (Supplementary Table 2) as well as general, additional barriers (Supplementary Table 3). Of the *n* = 17 responses, *n* = 7 were incomprehensible. It is noticeable that among the open responses, some barriers were mentioned which had already been listed in the closed-response format (e.g., time required, not wanting to disclose to other families). Barriers related to stigmatisation were mentioned most frequently (*n* = 4). This mainly concerned the association of depression with weakness (verbatim quotes: "signs of weakness", "weakness of one's personality" as well as the existence of depression only "in the minds of weak people") and the link between depression and "being crazy". Parents (*n* = 4) also stated that additional appointments for the child were undesirable: "their [children's] sports and friends are important to them and they don't want to take up more time," "son had a lot of appointments and we didn't want to add more," and "kids want to enjoy their free time after their school workload." In addition, numerous barriers were mentioned only once and there were some points mentioned, that were not included in the items with a closed response format.

*3) Is the impact of the barriers associated with current parental depression severity?* Table 3 shows the correlations between depression severity and the extent to which each barrier hindered parents from taking part in the prevention programme. The absolute correlations ranged from Min = 0.06 to Max = 0.51, and most of them were statistically significantly different from 0 (two-sided significance level: *p* < 0.0037).

**Table 3:** Correlation between depression severity and the extent to which certain factors are perceived as barriers

I don't want to be constantly reminded of my depression	.51	**
I feel ashamed of my depression	.48	**
I am concerned about negative effects on my child or children	.45	**
I see no connection between my mental health and the mental health of my child/children	.43	**
I feel too burdened by my depression	.42	**
I am afraid that I will be confronted with my own weaknesses during the study	.42	**
I do not want to talk to or open up in front of my child's father/mother	.41	**
I do not want to open up in front of other families	.41	**
I doubt that prevention can help	.40	**
I do not want to open up in front of my child or children	.35	**
I have not received enough information about the program	.35	**
I don't want my children to spend more time online than they already do	.33	**
My child or children refuse to participate	.32	**
The time commitment is too much for me	.31	**
My child is already receiving psychotherapy treatment	.30	**
My child/children do not need support	.28	**
I don't feel depressed anymore	-.28	*
I feel too burdened by the covid-19 pandemic	.27	**
I have feelings of guilt	.20	**
I have never been diagnosed with depression	.19	
In my culture, no one would participate in a prevention program like this	.18	
I am afraid that during my participation in the study it will become known that my children experience violence or are neglected by me	.18	
I want to avoid developing feelings of guilt	.14	
I do not want to receive psychotherapeutic support (anymore)	.13	
I think my child or children are too young to participate	.12	
I would never accept psychotherapeutic support	.10	
I am afraid that the other participants or the therapists involved will be prejudiced against me	.06	

Notes. **p < .0037 (two-sided significance level with Bonferroni correction)

#### Comparison of the sample with GuG-Auf-Online^1^ participants

In an exploratory post-hoc analysis, we descriptively compared the demographic characteristics of the survey sample with participants who took part in GuG-Auf-Online. There was a trend for the online survey parents to be younger than those who took part in the GuG-Auf-Online programme itself (see Fig. 1). In the survey sample there were fewer males (32%) than in GuG-Auf-Online (46%). Compared to GuG-Auf-Online participants, those who took part in the online survey had a somewhat lower level of education (see Fig. 2). Online survey participants had a higher proportion exhibited depressive symptoms (64% in the online survey, assessed via a self-administered questionnaire with cut-off, versus 35% in GuG-Auf-Online, assessed via a diagnostic interview. Survey participants had an average of *M* = 1.33 children (GuG-Auf-Online: *M* = 2.00 children) and 77% lived in a partnership (GuG-Auf-Online: 73% lived in a partnership). In total, 63% of the survey participants lived in a large city (at least 100,000 inhabitants; GuG-Auf-Online: 73%), 20% in a medium-sized city (20,000 to 100,000 inhabitants; GuG-Auf-Online: 16%), and the remaining participants came from small towns or rural communities. In summary, the survey participants tended to be younger, less educated and from smaller towns than those who participated in GuG-Auf-Online.

## Discussion

### Summary of the results

The overarching goal of the current study was to identify the possible barriers parents with a history of depression face to participating in the GuG-Auf-Online prevention programme. Overall, parents with depression were prevented from participating in GuG-Auf-Online by a range of different barriers. Six barriers had mean scores which indicated they prevented parents "quite a bit" or "a lot” from taking part in the programme: 1) overburden related to the covid-19 pandemic and 2) overburden due to depression, 3) not wanting to be reminded of one's depression, 4) feelings of shame due to the depression, 5) fear of confrontation with one's own weaknesses, and 6) not wanting to reveal oneself to other families. There was little evidence that the online setting was a barrier for parents. In free text responses, parents mostly mentioned stigmatization in a cultural context as well as barriers that were already included in the items with closed response format. For the majority of barriers, the extent to which parents were hindered by the barrier positively correlated with their current depression severity. This relates not only (a) to the desire not to be reminded of the depression, and (b) to the shame about their own depression, but also (c) to concern for their children. In summary, the more symptoms of depression parents were currently experiencing, the more they were discouraged from participating by concerns about negative effects on their child.

The six individual barriers identified can be grouped into the following themes: (a) overburden, (b) feelings of shame and (c) avoidance (e.g., to be reminded, confrontation, to open up). The survey participants tended to be younger, less educated and from smaller towns than those who participated in GuG-Auf-Online.

### Interpretation of the findings

In general, the finding that parents face a large number and variety of barriers to participating in a prevention programme for their children is consistent with previous studies of parents with depression [9, 25, 28].

Regarding overburden, many parents indicated that they were discouraged from participating in GuG-Auf-Online due to the stresses of the covid-19 pandemic. This was initially surprising, since during the period of the survey children’s leisure activities were generally available in Germany, schools were closed less often, psychotherapy sessions no longer had to take place online, and parents worked less from home than at the height of the covid-19 pandemic. However, the results of a representative cross-sectional study by Calvano, Engelke, Holl-Etten, Renneberg, and Winter [4] from December 2021 also show that in Germany, parents had poorer mental health during the period when data collection for the online survey began than during the first months of the covid-19 pandemic. These results support the finding from the online survey that parents were still feeling stressed by the covid-19 pandemic at the time of data collection.

The finding regarding the role of shame has been demonstrated elsewhere in regards to depression broadly (summarized by [14]) as well as specifically in the context of barriers to seeking prevention services for parents with depression [9, 25]. Shame thus seems to be a relevant issue for parents with depression seeking preventive support for their children and should therefore be carefully considered during recruitment and intervention efforts.

Regarding avoidance (not wanting to be reminded of the depression), previous literature has shown that the desire to avoid confrontation with one’s depression concerned parents whose depression was in remission [9, 28]. Our study extends these findings by showing that avoidance can also characterise parents who *currently* feel depressed. Furthermore, avoidance was the one barrier that correlated most highly with current symptoms of depression, suggesting that particularly parents who currently feel depressed may avoid participating in a programme like GuG-Auf-Online.

In addition to the three main themes described above, one additional barrier seems to be that parents did not see the connection between their own mental health and that of their children. This is consistent with one previous study [9]. In our sample, the lack of understanding about the connection between parental depression and the health of the children seems to be experienced as a barrier. Since a high proportion of participating parents in our sample felt depressed, it is conceivable that this finding (lack of understanding) could be related to the self-focus that often characterises people with depression. However, it is questionable as to what extent this finding can be generalised to all parents with depression because another interview study found that the majority of parents (with different diagnoses, but also depression) notice that their children are affected by parental illness (Stallard, Norman, Huline-Dickens, Salter, & Cribb, [29]).

Overall, many individual barriers seem to interact and contribute to parents not participating in GuG-Auf-Online.

### Implications for the implementation of GuG-Auf-Online

There are some implications that can be derived from the results of the online survey and our experiences with recruitment for GuG-Auf-Online and the online survey.

To improve advertising for GuG-Auf-Online and enhance the number of parents who participate in the programme, information available online about GuG-Auf-Online could be expanded. For example, it could be helpful to point out directly that many parents are reluctant to participate in such a prevention programme because of feelings of shame. It could help to signal to parents that we take these feelings seriously and that at the same time there is no reason to let these feelings hinder them. The employment of a participatory approach in the preparation of recruitment material might also be beneficial. This might include consulting parents who are affected by depression about how to best phrase informational material to avoid language perceived as stigmatizing or difficult to understand. Furthermore, there are plans to discuss with experts the extent to which potential participants can and should be given tools for dealing with shame in advance.

When it comes to mental illness, shame is often considered within the context of stigma [7, 11] and many interventions exist with the aim to reduce stigma in individuals affected by mental illness (e.g. [21]). Some of these intervention have implications that might also be useful in the context of preventive interventions. A study conducted by Alvidrez and colleagues (2009) included a sample of African Americans who were referred to outpatient mental health treatment. At the referral, half the sample was handed a psychoeducational booklet about stigma that was based on encouraging experiences of other African Americans who received mental health services. Their findings suggest that the psychoeducational booklet effectively reduced stigma in individuals with higher perceived treatment need and greater uncertainty about treatment [1]. Lu and colleagues [19] also report a reduction in depression-related stigma in patients of a community mental health clinic after receiving depression-specific psychoeducation. While the print version of the education was also effective, tablet-based multimedia education was found to have the greatest effect on stigma reduction (Lu et al., 2015). Therefore, when advertising preventive interventions such as GuG-Auf-Online it might be beneficial to provide psychoeducational material on how depression affects parents and families as well as encouraging testimonials of previous participants in recruitment material or as additional information online. The use of animated multimedia elements and videos might be especially effective in reducing stigma in parents affected by depression.

Regarding the overburden of parents, it would be helpful to point out in advance that whilst regular participation is a requirement of the programme, group leaders understand if participants need to skip individual sessions. Even though some parents have criticised the amount of time required to participate in GuG-Auf-Online, the authors are of the opinion that the current scope of the programme should be retained. Shortening the programme further may result in important content being removed and there may be too little time for exchange within one's own family and between families. An alternative might be to explicitly state that participation in the programme may reduce the levels of stress within the family in the long-term. The explorative (post-hoc) comparison of the survey sample with parents who took part in GuG-Auf-Online suggests that the latter tended to be older, had higher levels of education and lived in larger towns. Although caution is necessary when interpreting these findings due to the lack of apriori hypotheses and the different (but overlapping) time periods and methods of recruitment for the two samples, the findings suggest that the delivery of GuG-Auf-Online might reach a more heterogeneous sample if strategies to target younger and less-educated parents living in smaller towns are taken.

### Implications for other prevention programmes for parents

In general, when recruiting for similar prevention programmes, it might be helpful to focus on social networks and groups for parents. It might also be helpful to ask concerned parents to help with recruitment (e.g., forward flyers to other parents). The results of the survey indicate that the online setting is not necessarily a barrier to participation in prevention programmes. Especially for programmes where recruitment is difficult, it would be conceivable to increase the number of participants through an online setting. Regarding the barriers of potential participating parents, it is advisable to address feelings of shame (e.g., in the information material) and that there is no reason to let these feelings hinder parents. In this context, it would be helpful to draw potential participants' attention to the qualitative evaluation of GuG-Auf [5]: There, former participants reported that speaking publicly about their own depression was not as bad as they had feared. Furthermore, it might be helpful to offer opportunities for parents to talk with the study team about their personal barriers and to check whether their concerns really apply to the prevention programme.

### Strengths and limitations

The online survey conducted has some strengths and weaknesses. One clear strength is that the findings have important clinical implications not just for the implementation of Gug-Auf-Online specifically but also for family- and group-based prevention more generally. Not only did the findings replicate previous studies but the impact of novel barriers was also addressed. For example, to our knowledge it is the first study to show that the online setting is not an important barrier to participate in an prevention programme for parents with depression.

A second strength is the inclusion of a relatively large and young, not highly-educated sample of participants personally affected by depression. This group (young and not highly educated people) is often underrepresented in studies of prevention programs. Focusing on their views/attitudes could be helpful in attracting more families from this underrepresented group to prevention programmes. However, there was a trend for survey participants to be younger with a somewhat lower level of education (see Figs. 1 and 2 as well as the description of the sample) and a higher proportion exhibited depressive symptoms (64% in the online survey, assessed via a self-administered questionnaire with cut-off, versus 35% in GuG-Auf-Online, assessed via a diagnostic interview). An additional methodological strength was the inclusion not only of fixed-choice a-priori items but also open-ended questions in order to uncover additional barriers that were had not foreseen. In the open responses, only a few new aspects were mentioned, but predominantly those that had already been considered in the items with a closed response format. This indicates that the state of research is approaching theoretical saturation. What is still missing to reach theoretical saturation (that means a further data collection is no longer necessary as all relevant aspects have been collected), however, is the questioning of parents who fundamentally refuse to participate in prevention programmes that are tied into a research context. In the data available (in the open responses), there is no indication that such parents were included in our sample.

A category system with anchor examples was developed for the evaluation of the open response formats. The inter-coder reliability was very good and represents another strength of the study conducted.

Some weaknesses of the study are nonetheless worth mentioning. Firstly, it is unclear whether the negative wording of the items provoked negative response behaviour (in the sense of high item responses). This was not controlled for in the study. Secondly, it was difficult to understand the true meaning of some open-ended responses because they were very short. Future studies using interview or focus-group methodology may be better placed to extend our qualitative findings. Furthermore, unfortunately the cultural diversity of the sample is unclear. Although 28% of participants indicated that members of their cultural group would not participate in GuG-Auf-Online, it is not clear from the data which cultural groups these answers refer to. In hindsight, when constructing the items it would have been possible to use the *Perceived Barriers to Psychological Treatment Scale* (PBPT; [22]) as a guide. Although the scale measures barriers to *treatment*, there are overlaps in content with barriers to participation in prevention programmes. Finally, although descriptive findings suggest that the survey sample was more representative than the final sample of GuG-Auf-Online participants, differences between the samples in terms of the time period and methods of recruitment mean it was not possible to drawn robust conclusions about the extent to which demographic factors were a barrier to families taking part in GuG-Auf-Online.

### Directions for future research

The aim of the current study was to generate approaches for research questions that can be investigated in the future. The findings discussed above can be formulated in a research question as follows: Is there a positive correlation between the number of depressive symptoms and the extent to which parents are deterred from participating in a prevention program such as GuG-Auf-Online by their (a) feelings of shame, (b) overburden and (c) scepticism regarding the effectiveness and relevance of the prevention program?

These research question could be tested by studying a sample of parents with depression. The sample could complete two questionnaires: a questionnaire on current depression symptom severity and a questionnaire that measures the extent to which feelings of shame, overburden and scepticism keep them away from participating in a prevention programme. However, a questionnaire that measures the extent to which parents are deterred from participating in a prevention programme does not exist and would first have to be constructed. Here, it should be critically discussed how a questionnaire can be constructed to capture shame, overburden and avoidance so that the benefit of such a data collection justifies the effort of constructing a questionnaire.

Another line of research concerns parents' knowledge of the relationship between their depression and their children's health. The state of research on this to date is ambiguous. There are parents who are not aware of this connection. It should be investigated why some parents are not aware of this connection and whether it is advisable to make them aware of this connection in the interest of their children. A qualitative research approach with a mixed sample of affected persons and experts would be appropriate for this.

Lastly, future research might evaluate how the uptake of preventive family-based interventions is impacted by adding elements designed for stigma reduction to the recruitment material, such as animated psychoeducational material or encouraging testimonials from former participants.

## Conclusion

The aim of this study was to identify possible barriers faced by parents with depression to participating in the online preventive intervention GuG-Auf-Online. The findings of our online survey suggest that it is not so much features of the intervention itself (e.g. the online setting) that prevent affected parents from participating. Rather, it seems to be intrapsychic processes related to depression, e.g., avoidance, feelings of shame and being overburdened which contributed to their hesitance. For the successful implementation of future preventive programmes it might therefore be helpful to acknowledge and validate these feelings during the recruitment process using multi-media methods. For future recruitment for similar prevention programs, it may also be helpful to use social media advertisements and network distribution lists (in general for parents, not only for relatives of people with mental illness) to successfully reach younger parents with lower levels of education who do not necessarily live in large cities.

## Supplementary Information

Additional file1: Supplementary Table 1 Coding guide with anchor examples. Supplementary Table 2 Culture-related barriers. Supplementary Table 3.

## Data Availability

An anonymous version of the data file used for data analysis are available on the Open Science Framework here: https://osf.io/5z8bv/

## Abbreviatons

GuG-Auf (-Online): Growing Up Healthy and Happy (-Online)
FGCB: Family- and Group-based Cognitive-Behavioural Intervention
DASS: Depression-Anxiety-Stress Scales
PBPT: Perceived Barriers to Psychological Treatment Scale

## Footnotes

1 The data from GuG-Auf-Online are only related to the parent affected by depression or (if both parents were affected) considered the "affected parent" in the program.
