# Supplementary material for: Barriers to participating in an online family- and group-based prevention programme for parents with depression: an online survey
Source: BMC Psychol. 2025 Mar 19;13:275. doi: 10.1186/s40359-024-02266-8 (PMC11924649; doi:10.1186/s40359-024-02266-8)
Supplement: Supplementary file 1 — Additional file1: Supplementary Table 1 Coding guide with anchor examples. Supplementary Table 2 Culture-related barriers. Supplementary Table 3. [file 40359_2024_2266_MOESM1_ESM.docx]

Supplementary Table 1

*Coding guide with anchor examples*

| **Supercategory: Time** | |
| --- | --- |
| Expenditure of time | The time commitment keeps me away. |
| additional appointments for the child undesirable | My child already has enough appointments. I want my child to enjoy his free time. |
| Lack of time | I have too little time. |
| **Upper category: own burden/motivation** | |
| Burden due to own illness | My depression prevents me from participating. |
| Burden (unspecified) | Unclear statements such as "I have no strength" or "I am too burdened". |
| Laziness | I was too lazy. |
| **Upper category: negative effects** | |
| Avoiding negative effects within the family | Problems could arise within the family due to participation in GuG-Online. |
| Protection of the child from possible negative effects | I want to protect my child from negative effects. I do not want to burden my child. |
| **Upper category: Dealing with psychological problems** | |
| Culturally no exchange about depression | In my culture, people don't talk about depression. |
| Culturally psychological problems are not taken seriously | In my culture, mental health problems are not taken seriously. |
| Culturally lack of understanding of mental illness | In my culture there is no understanding of depression. |
| Avoidance of dealing with depression | I don't want to deal with the issue. |
| Not wanting to disclose to other families | I don't want to talk to other families. |
| Not wanting to disclose to own family | I don't want to talk about my depression with my family. |
| Stigmatization | Depression is associated with negative qualities. Depressed people are portrayed as crazy/weak/.... |
| Culturally related rejection of psychotherapy | In my culture, psychotherapy is seen as unnecessary. |
| **Upper category: online setting and content** | |
| No interest in online prevention | I have no interest in an online programme. |
| Doubts about effectiveness of online prevention | I am unsure if online programmes really help. |
| Fear: non-responsiveness to individual situation | I fear that GuG-Online cannot sufficiently address the individual needs within my family. |
| **Upper category: reasons/barriers that cannot be evaluated** | |
| Not understandable | Statements that are ambiguous or not understandable without further explanation/explanation, e.g. "prejudices" , "religion". |
| **Without upper categories** | |
| Language barriers | There are language barriers. |
| Parent sees no need | We don't need to participate because we are doing well. |
| Other parent would not participate in the programme | The other parent will not participate in the programme, e.g. because they don't have time, are not interested or don't know about the programme (no contact between parents). |
| Child not motivated | My child does not want to take part in such a programme. |
| Child in psychotherapeutic treatment | My child is already receiving psychotherapeutic treatment. |
| Difficulty in caring for child | I have small children whom I cannot leave alone. |

Supplementary Table 2

*Culture-related barriers*

| \|  \| *n* \| \| --- \| --- \| \| answer not understandable \| 6 \| \| ^a^ stigmatization \| 4 \| \| ^a^ culturally lack of understanding of mental illness \| 1 \| \| ^a^ culturally no exchange about depression \| 1 \| \| ^a^ culturally psychological problems are not taken seriously \| 1 \| \| ^a^ culturally related rejection of psychotherapy \| 1 \| \| ^a^ language barriers \| 1 \| \| expenditure of time \| 1 \| \| lack of time \| 1 \|   Notes. Wording of the item: “Are there any reasons that might prevent members of your cultural group from participating?” ^a^These categories have not yet been included in the closed-response items. Statements that were not understandable without further information were categorized as “not understandable” (e.g., short statements as “religion”). |  |
| --- | --- | --- | --- | --- | --- | --- | --- | --- | --- | --- | --- | --- | --- | --- | --- | --- | --- | --- | --- | --- | --- |

Supplementary Table 3

*Other barriers that keep parents away from participating*

|  | *n* |
| --- | --- |
| additional appointments for the child undesirable | 4 |
| ^a^ burden (unspecified) | 3 |
| expenditure of time | 3 |
| child in psychotherapeutic treatment | 2 |
| Answer not understandable | 1 |
| protection of the child from possible negative effects | 2 |
| lack of time | 2 |
| ^a^ fear: non-responsiveness to individual situation | 1 |
| burden due to own illness | 1 |
| parent sees no need | 1 |
| ^a^ laziness | 1 |
| ^a^ no interest in online prevention | 1 |
| child not motivated | 1 |
| ^a^ difficulty in caring for children | 1 |
| not wanting to disclose to other families | 1 |
| not wanting to disclose to own family | 1 |
| avoidance of dealing with depression | 1 |
| ^a^ avoiding negative effects within the family | 1 |
| ^a^ doubts about effectiveness of online prevention | 1 |
| ^a^ other parent would not participate in the programme | 1 |

Notes. Wording of the item: “Are there any other reasons or possible barriers that might prevent you from participating in the study?”
^a^These categories have not yet been included in the closed-response items. A statement that was not understandable without further information was categorized as “not understandable”.
